# Schooling and Covid-19: lessons from recent research on EdTech

**DOI:** 10.1038/s41539-020-00072-6

**Published:** 2020-08-11

**Authors:** Robert Fairlie, Prashant Loyalka

**Affiliations:** 1 Department of Economics, University of California, Santa Cruz, USA; 2 Graduate School of Education/Freeman Spogli Institute for International Studies, Stanford University, Stanford, USA

**Keywords:** Education, Economics

## Abstract

The wide-scale global movement of school education to remote instruction due to Covid-19 is unprecedented. The use of educational technology (EdTech) offers an alternative to in-person learning and reinforces social distancing, but there is limited evidence on whether and how EdTech affects academic outcomes. Recently, we conducted two large-scale randomized experiments, involving ~10,000 primary school students in China and Russia, to evaluate the effectiveness of EdTech as a substitute for traditional schooling. In China, we examined whether EdTech improves academic outcomes relative to paper-and-pencil workbook exercises of identical content. We found that EdTech was a perfect substitute for traditional learning. In Russia, we further explored how much EdTech can substitute for traditional learning. We found that EdTech substitutes only to a limited extent. The findings from these large-scale trials indicate that we need to be careful about using EdTech as a full-scale substitute for the traditional instruction received by schoolchildren.

The wide-scale global movement of school education to remote instruction due to Covid-19 is unprecedented. The use of educational technology (EdTech) offers an alternative to in-person learning and reinforces social distancing, but there is limited evidence on whether and how EdTech affects academic outcomes, and that limited evidence is mixed.^1,2^ For example, previous studies examine performance of students in online courses and generally find that they do not perform as well as in traditional courses. On the other hand, recent large-scale evaluations of supplemental computer-assisted learning programs show large positive effects on test scores. One concern, however, is that EdTech is often evaluated as a supplemental after-school program instead of as a direct substitute for traditional learning. Supplemental programs inherently have an advantage in that provide more time learning material.

Recently, we conducted two large-scale randomized experiments, involving ~10,000 primary school students in China and Russia, to evaluate the effectiveness of EdTech as a substitute for traditional schooling.^3,4^ In both, we focused on whether and how EdTech can substitute for in-person instruction (being careful to control for time on task). In China, we examined whether EdTech improves academic outcomes relative to paper-and-pencil workbook exercises of identical content. We followed students ages 9–13 for several months over the academic year. When we examined the impacts of each supplemental program we found that EdTech and workbook exercise sessions of equal time and content outside of school hours had the same effect on standardized math test scores and grades in math classes. As such, EdTech appeared to be a perfect substitute for traditional learning.

In Russia, we built on these findings by further exploring how much EdTech can substitute for traditional learning. We examined whether providing students ages 9–11 with no EdTech, a base level of EdTech (~45 min per week), and a doubling of that level of EdTech can improve standardized test scores and grades. We found that EdTech can substitute for traditional learning only to a limited extent. There is a diminishing marginal rate of substitution for traditional learning from doubling the amount of EdTech use (that is, when we double the amount of EdTech used we do not find that test scores performance doubles). We find that additional time on EdTech even decreases schoolchildren’s motivation and engagement in subject material.

The findings from the large-scale trials indicate that we need to be careful about using EdTech as a full-scale substitute for the traditional instruction received by schoolchildren. There are two general takeaways: First, to a certain extent, EdTech can successfully substitute for traditional learning. Second, there are limits on how much EdTech may be beneficial. Admittedly, we need to be careful about extrapolating from the smaller amount of technology substitution in our experiments to the full-scale substitution in the face of the coronavirus pandemic. However, these studies may offer important lessons. For example, a balanced approach to learning in which schoolchildren intermingle work on electronic devices and work with traditional materials might be optimal. Schools could mail workbooks to students or recommend that students print out exercises to break up the amount of continuous time schoolchildren spend on devices. This might keep students engaged throughout the day and avoid problems associated with removing the structure of classroom schedules. Schools and families can devise creative remote learning solutions that include a combination of EdTech and more traditional forms of learning. Activities such as reading books, running at-home experiments, and art projects can also be used to break up extensive use of technology in remote instruction.
